# Non-coding RNAs in Rheumatoid Arthritis: From Bench to Bedside

**DOI:** 10.3389/fimmu.2019.03129

**Published:** 2020-01-28

**Authors:** Jinghua Wang, Shushan Yan, Jinghan Yang, Hongying Lu, Donghua Xu, Zengyan Wang

**Affiliations:** ^1^Clinical Medicine College, Weifang Medical University, Weifang, China; ^2^Department of Rheumatology, The Affiliated Hospital of Weifang Medical University, Weifang, China; ^3^Department of Gastrointestinal and Anal Diseases Surgery, The Affiliated Hospital of Weifang Medical University, Weifang, China; ^4^Functional Laboratory, Clinical Medicine College of Weifang Medical University, Weifang, China; ^5^Department of Operating Room, Zhucheng People's Hospital, Zhucheng, China

**Keywords:** circRNA, exosome, lncRNA, microRNA, non-coding RNA, rheumatoid arthritis

## Abstract

Rheumatoid arthritis is a common systemic and autoimmune disease characterized by symmetrical and inflammatory destruction of distal joints. Its primary pathological characters are synovitis and vasculitis. Accumulating studies have implicated the critical role of non-coding RNAs (ncRNAs) in inflammation and autoimmune regulation, primarily including microRNA (miRNA), long non-coding RNA (lncRNA), and circular RNA (circRNA). NcRNAs are significant regulators in distinct physiological and pathophysiological processes. Many validated non-coding RNAs have been identified as promising biomarkers for the diagnosis and treatment of RA. This review will shed some light on RA pathogenesis and be helpful for identifying potential ncRNA biomarkers for RA.

## Introduction

Rheumatoid arthritis (RA) is a type of chronic autoimmune disease, characterized by synovitis and vasculitis in pathology. It is a highly disabling disease due to joint deformity and loss of function (1). The main clinical features of RA typically are symmetrical polyarthritis with distal joint redness, swelling, and pain, especially the small joints of hands and feet (2). Approximately 1% of the population is affected with RA worldwide, with a higher prevalence in Europeans and Asians (3). Studies have implicated the significant and complex roles of genetic factor and environmental factor in the etiology of RA (4, 5). It has been well-documented that inflammatory response and immunological disorders critically contribute to RA. However, the precise pathogenesis and etiology of RA remain to be completely elucidated (6). To the best of our knowledge, common laboratory tests used for RA generally include erythrocyte sedimentation rate (ESR), c-reactive protein (CRP), rheumatoid factor (RF), and anti-cyclic peptide containing citrulline (anti-CCP) antibodies (7). Nevertheless, they lack specificity and have low priority. As a result, identification of novel and promising biomarkers for RA is essential for its early diagnosis and treatment.

In human, non-protein coding genes occupy ~70% of the genome. Accumulating data have suggested non-coding RNAs (ncRNAs) play important roles in regulating autoimmunity and inflammation (8). Due to increasing development of microarray sequencing techniques and bioinformatics analysis, many ncRNAs have been identified and validated in many kinds of diseases (9–12). They can be regarded as promising biomarkers predicting the occurrence and progression of cancer, cardiovascular disease and autoimmune disease, and so on (9–12). Different autoimmune disease has different ncRNA expression profile in diverse cells and tissues. In addition, there are still some ncRNAs dysregulated in several kinds of inflammatory or autoimmune diseases with similarities. Accumulating studies have suggested some ncRNAs are specifically expressed in RA, mainly including microRNAs (miRNAs), long non-coding RNAs (lncRNAs), and circular RNAs (circRNAs) (7, 13, 14). Previously, we have identified the specific profile of miRNAs and lncRNAs differentially expressed in RA, which can serve as promising markers for RA diagnosis and treatment (15–17). Nonetheless, the modifying effects and molecular mechanism of those specifically expressed ncRNAs in RA pathogenesis have not been fully elucidated up to date.

In the present study, some functional ncRNAs have been listed in Table 1. The potential targets and mechanisms of them are also summarized. We aim to focus on the current knowledge of ncRNAs in RA, primarily including miRNAs, lncRNAs, and circRNAs by reviewing all currently published studies. Clarification of the expression and molecular mechanism of dysregulated ncRNAs in inflammation and autoimmunity will help to understand the pathogenesis of RA. Most importantly, identifying the targeted genes of those aberrantly expressed ncRNAs in RA will be useful for investigating promising biomarkers for its early diagnosis and efficient treatment.

**Table 1:** Aberrant expressed ncRNAs in RA.

**NcRNAs**	**Target**	**Site**	**Expression**	**Signaling**	**References**
**MiRNA**					
miR-548a-3p	TLR4	Serum, PBMC	Down	TLR4/NF-κB signaling	Wang et al. (15)
miR-6089	TLR4	Serum, PBMC	Up	TLR4 signaling	Xu et al. (16)
miRNA-150-5p	MMP14/VEGF	Mesenchymal cell-derived exosomes	Down	Unknown	Chen et al. (18)
miR-338-5p	NFAT5	Synoviocytes	Up	Unknown	Guo et al. (19)
miR-708-5p	Unknown	Synoviocytes	Down	Wnt3a/β-catenin pathway	Wu et al. (20)
miR-143-3p	IGF1R/IGFBP5	Synovium tissues	Up	Ras/p38 MAPK signaling	Yang et al. (21)
miR146a/b	Unknown	Peripheral blood and joint tissues	Up	Unknown	Churov et al. (22)
miR155	Unknown	Peripheral blood and joint tissues	Up	Unknown	Churov et al. (22)
miR16	Unknown	Peripheral blood and joint tissues	Up	Unknown	Churov et al. (22)
miR223	Unknown	Peripheral blood and joint tissues	Up	Unknown	Churov et al. (22)
**LncRNA**					
RNA143598	Unknown	Serum	Up	Unknown	Xu et al. (17)
RNA143596	Unknown	Serum	Up	Unknown	Xu et al. (17)
HIX0032090	lncRNA-mRNA network	Serum	Up	NF-κB signaling	Xu et al. (17); Yan et al. (23)
IGHCγl	Unknown	Serum	Up	Unknown	Xu et al. (17)
XLOC-002730	Unknown	Serum	Up	Unknown	Xu et al. (17)
H19	Unknown	Synovium tissues	Up	MAPK/PI3K pathway	Stuhlmuller et al. (24)
LincRNA-p21	RELA	Peripheral blood	Down	NF-κB/PKcs signaling	Spurlock et al. (25)
C5T1lncRNA	C5	PBMC and tissues	Up	Unknown	Messemaker et al. (26)
LOC100652951	Unknown	T cells	Up	Unknown	Lu et al. (27)
LOC100506036	SMPD1/NFAT1	T cells	Up	Unknown	Lu et al. (27)
LncRNANTT	PBOV1	Monocyte/macrophage	Up	NTT/PBOV1 axis	Yang et al. (28)
HOTAIR	miR-138	Chondrocytes	Down	NF-κB signaling	Zhang et al. (29)
lncRNA S5645.1	miR-152/miR-20	Peripheral blood and tissues	Down	Unknown	Jiang et al. (30)
lncRNA XR_006437.1	XR_006437.1-miRNA-mRNA network	Peripheral blood and tissues	Down	Unknown	Jiang et al. (30)
lncRNA J01878	J01878-miRNA-mRNA network	Peripheral blood and tissues	Down	Unknown	Jiang et al. (30)
lncRNA GAPLINC	miR-382-5p/miR-575	Fibroblast-Like synoviocytes	Up	GAPLINC-related pathways	Mo et al. (31)
ZFAS1	miR-27a	Fibroblast-Like synoviocytes	Up	Unknown	Ye et al. (32)
**CircRNA**					
circ_102594	circRNA-miRNA ceRNA network	PBMC	Down	Unknown	Zheng et al. (14)
circ_103334	circRNA-miRNA ceRNA network	PBMC	Up	Unknown	Zheng et al. (14)
circ_104194	circRNA-miRNA ceRNA network	PBMC	Up	Unknown	Zheng et al. (14)
circ_104593	circRNA-miRNA ceRNA network	PBMC	Up	Unknown	Zheng et al. (14)
circRNA_003524	Unknown	PBMC	Up	Unknown	Ouyang et al. (33)
circRNA_103047	Unknown	PBMC	Up	Unknown	Ouyang et al. (33)
circRNA_104871	Unknown	PBMC	Up	Unknown	Ouyang et al. (33)
circRNA_101873	Unknown	PBMC	Up	Unknown	Ouyang et al. (33)
circ_0001859	ATF2	Synovium tissues	Up	miR-204/211/ATF2	Li et al. (34)

## MiRNAs

MiRNAs are evolutionarily conserved and usually have a length of 18–25 nucleotides, which regulate the expression of targeted genes at the post-transcriptional level by promoting the degradation of mRNA or repressing its translation (7). Accumulated studies have suggested the critical role of miRNAs in several kinds of autoimmune diseases, such as systemic lupus erythematosus (SLE), RA and Sjögren's syndrome (35). However, the expression and function of those aberrantly expressed miRNAs may be different in diverse autoimmune diseases. MiRNAs play a pivotal role in the regulation of multiple physiological and pathological processes, including cell cycle, stem cell maintenance, organ development, angiogenesis, and carcinogenesis (36). A number of well-established miRNAs have been regarded as candidate biomarkers for RA due to their critical role in regulating inflammation and autoimmunity (37). They are widely expressed in various cells, tissues, or microsomes and contribute to the pathogenesis of RA (37). Besides, some miRNAs are differentially expressed in response to TNF inhibitor treatment and other conventional therapies (38). Accordingly, miRNA can serve as predictive factor for the clinical response to biological therapies among RA patients.

**Figure 1:**
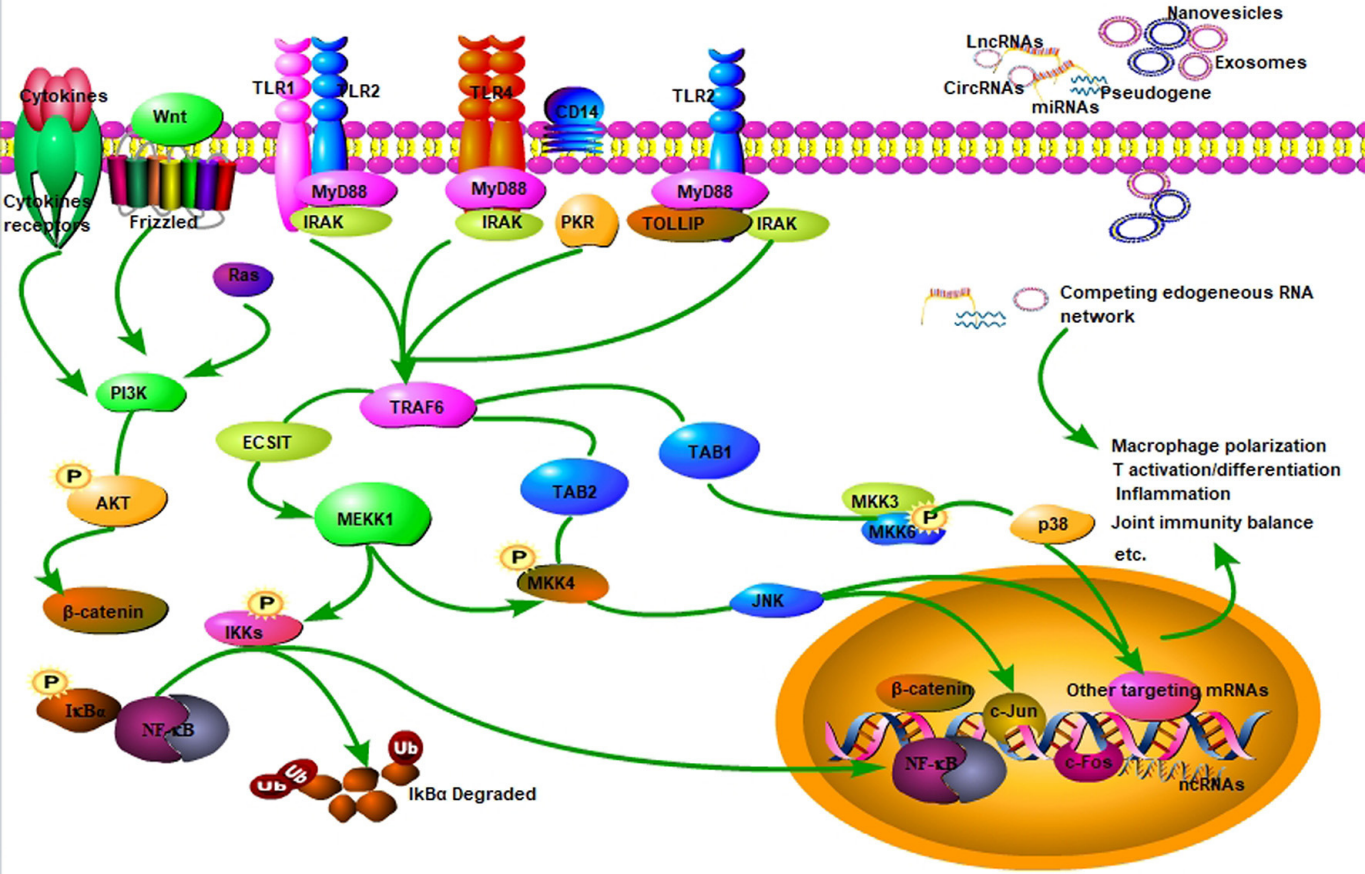
Signaling pathway of ncRNAs in RA. NcRNAs (miRNA, lncRNA, circRNA) are involved in regulating inflammation and autoimmunity, such as immune cell activation, differentiation, and polarization. Some ncRNAs are encapsulated in nanovesicles and exert critical effects on inflammatory and immune cells, and some can function as ceRNA by sponging miRNAs in RA. NcRNAs participate in RA inflammation and autoimmune disorders primarily through TLR4/NF-κB, MAPK/PI3K, and Wnt3a/β-catenin signaling pathways, and so on.

As shown in Table 1, a variety of miRNAs are differentially expressed and dysregulated in RA, which can negatively regulate targeted genes, such as those genes encoding cytokines, chemokines, and inflammation-related signaling molecules, and thus participate in the pathogenesis of RA (22, 39, 40). Moreover, it has been well-established some nanovesicles-delivered miRNAs specially expressed in RA and exert modifying effects on inflammation and autoimmunity, such as exosomes-encapsulated miRNAs (15, 16). Exosomes are cell-derived vesicles encapsulating functional molecules such as RNAs, DNAs, proteins, and lipids (41, 42). Exosomes usually mediate intracellular communication by delivering functional RNAs from donor to receipted cells, including ncRNAs of miRNAs, lncRNAs as well as circRNAs (Figure 1). Mounting data have implicated exosomes and their encapsulated functional ncRNAs have been recognized as potential biomarkers for RA, especially exosome-encapsulated miRNAs (16, 18, 43).

Growing data have revealed that many free miRNAs and exosome-delivered miRNAs are closely associated with RA (44, 45). The molecular mechanism of differentially expressed miRNAs in RA has been widely investigated by many published studies, particularly regarding their altering effects on inflammation and autoimmunity (15, 46–48). Toll-like receptors (TLRs), such as TLR2 and TLR4, are vital pattern recognition receptors (PRRs) functioning as a bridge linking immunomodulation and inflammatory response in many autoimmune diseases, including RA (35, 49, 50). Mechanisms of different TLRs in immune and inflammatory cells have been extensively investigated (Figure 1). Our previous study has demonstrated that miR-6089 inhibits inflammatory response via targeting TLR4 (16). It has been well-documented signaling pathways of TLRs/NF-κB, cytokines, and chemokines as well as Wnt signal play vital roles in regulating inflammatory response and immunological reaction that are involved in RA pathogenesis (Figure 1) (15, 46–48). The study by Guo et al. has shown that the proliferation, apoptosis and migration of fibroblast-like synoviocytes in RA can be affected by miR-338-5p via targeting NFAT5 (19). MiR-708-5p can promote the apoptosis of fibroblast-like synoviocytes and alleviate RA through Wnt3a/β-catenin pathway (20). MAPK signaling is also well-documented in regulating miRNAs in RA (Figure 1) (21, 46, 51). Our previous study has demonstrated that exosome-delivered miR-548a-3p regulates macrophages-mediated inflammation through TLR4/NF-κB signaling pathway in RA (15). Therefore, miR-548a-3p may serve as a promising marker for RA, because it can alleviate inflammation in RA. The miR-548a-3p/TLR4/NF-κB axis will offer new therapeutic strategies for RA. Taken together, the differentially expressed miRNAs in peripheral circulation or extracellular vesicles or synovium tissues in RA would be identified as important biological targets for the diagnosis and treatment of RA patients. Nevertheless, more pre-clinical or clinical experiments are warranted for more investigations.

## LncRNAs

LncRNA is a newly identified non-coding RNA widely expressed in various tissues of the human body, which consists of more than 200 nucleotides in length (17). According to the structure and function of lncRNA, lncRNAs can be divided into five categories: sense, antisense, bidirectional, intronic, and intergenic (52). Some lncRNAs exert oncogenic properties in cancer (53), while some can inhibit the development and progression of malignancies due to distinct expression and biological effects in cancer cells (54). Accumulating studies have implicated that a variety of lncRNAs are found to be differentially expressed and confer effects on immune cells in several kinds of autoimmune diseases, including RA (55–58). Different autoimmune diseases have specific lncRNA expression profiles, which may be also specifically expressed in different cells and tissues. Besides, the lncRNA expression profile in RA can be influenced by different therapy strategies demonstrated by Guo et al. (59). Furthermore, it has been reported that a number of lncRNAs are dysregulated and associated with organ damage in systemic lupus erythematosus (SLE) compared with RA (60), which suggests a critical role of ncRNA in regulating specific organ damage in autoimmune diseases. LncRNA H19, Hotair, lincRNA-p21, C5T1, LOC100652951, and LOC100506036 have been verified to be dysregulated in T cells, peripheral blood mononuclear cells (PBMCs), exosomes, and synovial cells in RA, which are associated with inflammation and immune reaction in RA (Table 1) (24–27, 61). The lncRNA expression profile in RA is different in diverse types of immune cell, such as B cells, nature killer (NK) cells, and T cells, which suggests immune cell-type specificity of lncRNA expression (62). Identification of aberrantly expressed lncRNAs in RA and exploration of the underlying molecular mechanisms will offer a new direction to understand the pathogenesis of RA.

The regulatory mechanism of lncRNAs is complicated and needs to be investigated by more functional and mechanical experiments. T lymphocytes-mediated autoimmune response plays an important role in the development of RA (63, 64). Moreover, the abnormally expressed lncRNAs in T cells can influence their function and facilitate or suppress immune and inflammatory reactions in RA, such as lncRNA FAM66C, LOC100652951, and LOC100506036 (27, 64, 65). PBMC and exosome-derived Hotair are demonstrated to affect the migration of activated macrophages and the expression of MMP-2 and MMP-13 in RA (61). An lncRNA NTT/PBOV1 axis has been elucidated by a published study, which is capable of regulating monocyte differentiation in RA (28). LncRNA HOTAIR is documented to alleviate RA by targeting miR-138 and inhibit the activation of NF-κB pathway in LPS-treated chondrocytes, suggesting an lncRNA-miRNA interaction in RA pathogenesis (29). In our previous study, five lncRNAs are reported to be significantly up-regulated in serum samples of RA patients, including RNA143598, RNA143596, HIX0032090, IGHCγl, and XLOC-002730 (17) (Table 1). Some of these aberrantly expressed lncRNAs are associated with the disease course, anti-CCP antibody level and disease activity of RA (17). The bioinformatics analysis indicates that classic signaling pathways of TLRs, cytokines, NF-κB, and IRF3/IRF7 that are most likely involved in RA with regard to lncRNAs regulation (17). More interestingly, HIX0032090 has been demonstrated to participate in RA pathogenesis by functioning as a competitive endogenous RNA (ceRNA) for miRNA in our recently published study (23). Nevertheless, more future studies are warranted to elucidate the molecular mechanism of those dysregulated lncRNAs in RA initiation and progression. Taken together, these available data have suggested the immune cell specificity of lncRNA expressed in RA.

**Figure 2:**
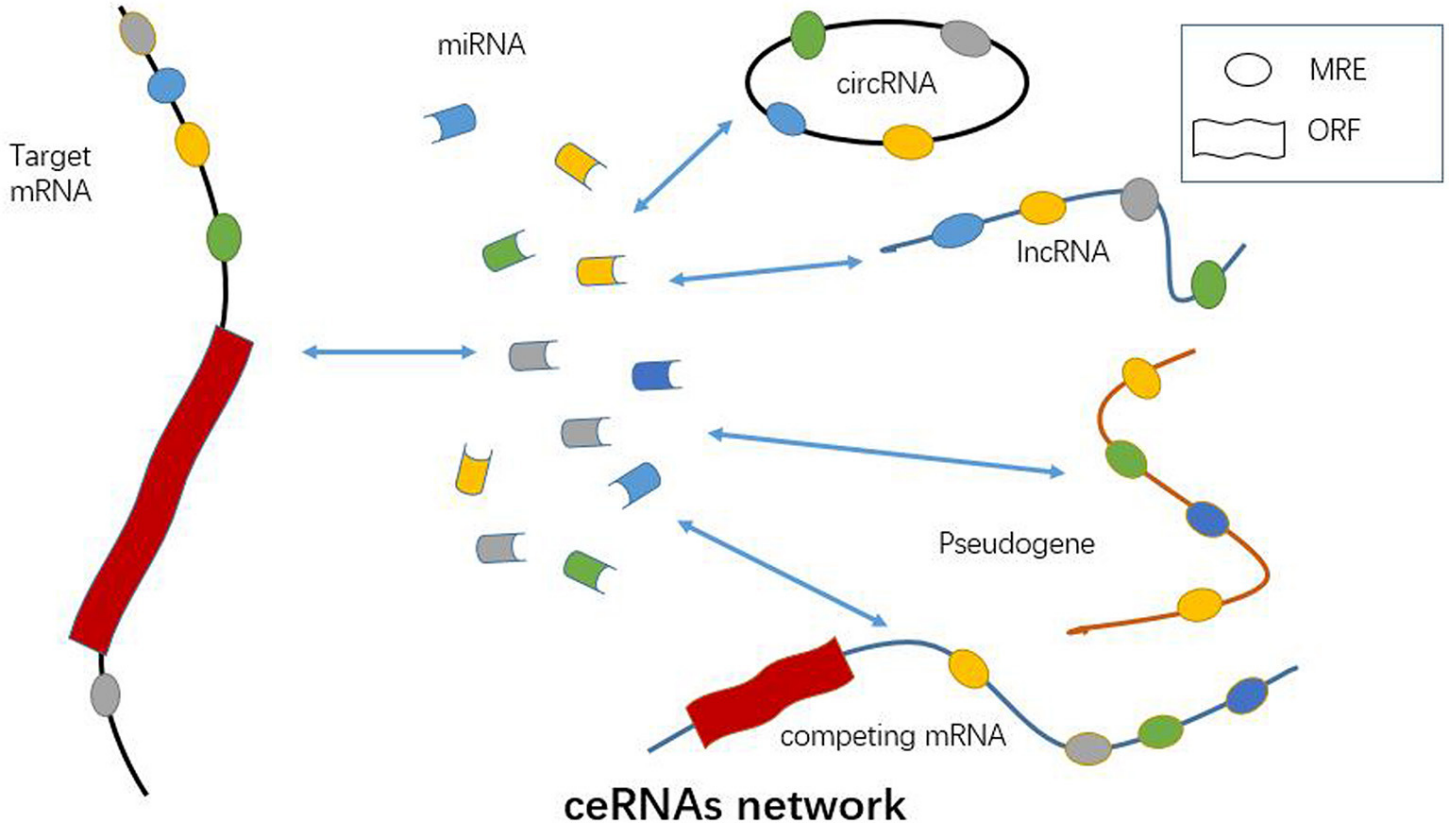
Functional role of ncRNAs as ceRNA. A number of miRNAs, lncRNAs, circRNAs, pseudogenes, and competing mRNAs can act as ceRNA and promote the expression of targeted mRNAs via sponging miRNAs. CeRNA network is a crucial mechanism of ncRNAs involved in RA pathogenesis.

Mounting evidence has suggested lncRNA, the same as pseudogenes, circRNAs and competing mRNAs, can function as ceRNA based on a lncRNA-miRNA-mRNA network in autoimmune disease, vascular disease, cancer, and so on (Table 1 and Figure 2) (66–70). LncRNA may facilitate the expression and function of the targeted mRNA by sponging miRNA, and thus participates in regulating immune cell activity and function (71). Jiang et al. have found that three lncRNAs, namely S5645.1, XR_006437.1 and J01878, can serve as promising biomarkers for RA via ceRNA network (30). It has also been demonstrated that lncRNA GAPLINC enhances cell proliferation, migration, and generation of proinflammatory cytokines by sponging miR-382-5p and miR-575 in fibroblast-like synoviocytes (31). Similarly, ZFAS1, a newly identified lncRNA in RA, is shown to modulate fibroblast-like synoviocytes migration and invasion by targeting miR-27a as a sponge (32). As mentioned above, an lncRNA HOTAIR-miR-138-NF-κB axis has also been established in chondrocytes in RA (29). Accordingly, lncRNA may function through ceRNA mechanism by sponging one or more miRNAs in immune cell or parenchymal cell, such as chondrocytes (Figure 2). Identification of lncRNA-miRNA-mRNA ceRNA network provides new insight into the pathogenesis of RA. Key molecules and signaling pathway in this network will serve as ideal diagnostic and therapeutic targets for RA.

## CircRNAs

Circular RNA (circRNA) is an endogenous non-coding RNA, the most representative characteristic of which is the covalently closed RNA circle without 5′ end caps or 3′ poly (1) tails (33, 72). This circular structure is usually stable with the half-life larger than 48 h (73). CircRNAs are primarily divided into three types of circRNA including exonic circRNAs (ecircRNAs), circular intronic RNAs (ciRNAs), and exon-intron circRNAs (EIciRNAs) (74). The production of circRNAs in cells is usually attributed to exon skipping and circularization driven by intron pairing or RNA binding protein (74). Apart from mammals, numerous circRNAs have been demonstrated to be expressed in fungi, plants, and protists (75–78). Most importantly, the expression of circRNAs is in a tissue-specific manner (79). Usually, circRNAs can be found in peripheral blood, exosomes, and tissues. Similar to lncRNA, circRNA can also serve as miRNA sponge, which can combine with miRNAs and thereby insulate them from the natural mRNAs (70, 80–83) (Figure 2). Available data have revealed ecircRNA confers critical effects on several pathological and physiological processes mainly through ceRNA mechanism in cytoplasm (74). However, circRNAs of ciRNAs and EIciRNAs usually regulate the targeted genes in nucleus (84–86). CircRNAs serve as miRNAs sponge and facilitate the expression of targeted mRNAs by inhibiting the effect of miRNA (82, 87). CeRNA is also an essential way for circRNA in regulations of autoimmunity and inflammation (88, 89) (Figure 2). However, there are few publications elucidating the ceRNA regulatory mechanism of circRNA in RA up till now.

CircRNAs are suggested in regulating diverse immune disorders due to their various forms of epigenetic modification, for instance, miRNA sponge and miRNA reservoir (74, 79, 83). Accumulated data have implicated the vital role of circRNAs in multiple kinds of diseases, such as cancer, neurologic disorders and cardiovascular diseases (74, 90–92). The critical role of circRNAs in antiviral immunity has been well-documented, which offers potential therapeutic strategies for antiviral therapy by targeting circRNAs (93–95). The study by Ma et al. shows the evidence that circARSP91 promotes cancer immune surveillance by regulating NK cells in liver cancer, suggesting a critical role of circRNA in tumor immunity (96). In addition, circRNA Malat-1 has been suggested as a key regulator in alloimmune rejection by promoting dendritic cells to induce T cell exhaustion and regulatory T cell generation, which implicates the pivotal role of circRNA in adaptive immunity (97). Taken together, circRNA plays critical roles not only in innate immunity but adaptive immunity.

During the past few years, the role of circRNAs in RA has drawn more and more attention. There is specific circRNA expression profile in RA as demonstrated by microarray chip analysis (14, 33, 98). As shown in Table 1, many circRNAs have been documented to be aberrantly expressed in RA, such as circRNA_092516, circRNA_003524, circRNA_103047, and circRNA_101873. CircRNAs can be up-regulated or down-regulated in peripheral blood or tissues in RA. Interaction between miRNA and circRNA is also revealed in RA, which implicates the circRNA-miRNA network in autoimmune regulation (99). It has been shown that has-circ-0001859 is identified in synovial tissues, which regulates synovial inflammation via sponging miR-204/211 and targeting ATF1 (34). Accordingly, circRNAs can regulate RA through ceRNA network (Figure 2). Nevertheless, little is known about the downstream signaling pathway of circRNA in regulating autoimmunity and inflammation. More studies are warranted to elucidate this issue in future. It is also prospective to investigate novel diagnostic and therapeutic strategies for RA by targeting circRNAs.

## Conclusions and Future Directions

In the last few years, ncRNAs have been regarded as hot points in many scientific fields worldwide. Role of ncRNAs in regulating inflammation and autoimmunity has drawn widely attention. Although specific expression profiles of miRNAs, lncRNAs and circRNAs in RA have been well-documented in many currently published studies, the molecular mechanism behind ncRNAs regulation in RA is not very clear yet. Those aberrantly expressed ncRNAs participate in the pathogenesis of RA primarily by regulating autoimmunity and inflammation. Up to now, Wnt3a/β-catenin, TLR/NF-κB, and MAPK signaling pathways have been well-established in regulating the differentially expressed ncRNAs in RA. Most interestingly, elucidation of the lncRNA/circRNA-miRNA-mRNA ceRNA network sheds light on the pathogenesis of RA. Researchers are encouraged to investigate novel strategies for the early diagnosis and treatment of RA by targeting ncRNAs and relevant key signaling pathways in the future.

## Author Contributions

JW, SY, HL, and JY carried out literature research and reviewed all articles. JW, SY, and HL wrote the paper. ZW and DX edited the article.

### Conflict of Interest

The authors declare that the research was conducted in the absence of any commercial or financial relationships that could be construed as a potential conflict of interest.
